# *mcr*-Positive *Escherichia coli* ST131-*H*22 from Poultry in Brazil

**DOI:** 10.3201/eid2608.191724

**Published:** 2020-08

**Authors:** Andre Becker S. Saidenberg, Marc Stegger, Lance Bradley Price, Thor Bech Johannesen, Maliha Aziz, Marcos P.V. Cunha, Andrea M. Moreno, Terezinha Knöbl

**Affiliations:** University of São Paulo, São Paulo, Brazil (A.B.S. Saidenberg, M.P.V. Cunha, A.M. Moreno, T. Knöbl);; George Washington University, Washington, DC, USA (M. Stegger, L.B. Price, M. Aziz);; Statens Serum Institut, Copenhagen, Denmark (M. Stegger, T.B. Johannesen)

**Keywords:** Antimicrobial resistance, avian pathogenic *Escherichia coli*, Brazil, colistin, *Escherichia coli*, MDR, phylogenetic analyses, poultry, ST131, virulence factors, zoonoses

## Abstract

*Escherichia coli* sequence type (ST) 131 is of concern because it can acquire antimicrobial resistance and cause extraintestinal infections. *E. coli* ST131-*H*22 sublineage appears capable of being transmitted to humans through poultry. We report on multidrug-resistant ST131-*H*22 poultry isolates in Brazil closely related to international human and poultry isolates.

The pandemic, extraintestinal, pathogenic *Escherichia coli* multilocus sequence type (MLST) 131 lineage has emerged extensively, gaining notoriety for its extensively multidrug-resistant ST131-*H*30 sublineage (*1*). Whereas ST131-*H*30 appears to be transmitted primarily from person to person, the *H*22 sublineage may be transmitted zoonotically through poultry and cause urinary tract infections and urosepsis (*2*,*3*). We report isolating ST131-*H*22 strains that are multidrug resistant (MDR), meaning that they are resistant to >3 classes of antimicrobials (*4*), carrying mobile colistin-resistance (*mcr*) determinants from poultry in Brazil, the largest poultry-exporting country in the world. 

We collected 64 *E. coli* strains from poultry with colibacillosis cases from 2 different farms in the same geographic region of Brazil and screened them by PCR for the ST131 clonal group (*5*). PCR detected 6 ST131 isolates (2 from the first farm, 4 from the second), which we whole-genome sequenced (BioProject no. PRJNA398035). We determined phenotypic antimicrobial susceptibility with disk diffusion testing, except for isolates carrying the *mcr* gene, which we tested using broth microdilution (*6*).

We trimmed the reads and used QUAST (http://quast.sourceforge.net) to evaluate the quality of assemblies (contig lengths and expected genome sizes). We assembled DNA sequences with SPAdes (http://cab.spbu.ru/software/spades), then determined the serotype, phylogroup, MLST, *fimH* protein type, virulence gene profile, plasmid replicons, and markers of antimicrobial resistance for each isolate *in silico* using the ABRicate virulence factors database (https://github.com/tseemann/abricate) and ResFinder/PlasmidFinder tools from CGE (https://cge.cbs.dtu.dk/services). Genes were identified with a minimum of >95% of identity and coverage.

We identified all isolates as O25:H4-ST131-*H*22, all belonging to phylogroup B2. We generated a maximum-likelihood phylogeny tree on the basis of core-genome single-nucleotide polymorphisms, including the 6 isolates from Brazil and 140 ST131-*H*22 sequences from EnteroBase (http://enterobase.warwick.ac.uk) and a previous study (*2*), using the Northern Arizona SNP Pipeline (https://tgennorth.github.io/NASP/) aligned against *E. coli* JJ1886 ST131-*H*30 (GenBank accession no. CP006784) (Appendix). The 6 isolates from poultry were nested within a clade of intermingled poultry and human clinical isolates within the overall international isolates (Figure, panel A). The isolates from Brazil were closely related to ST131-*H*22 avian pathogenic *E. coli* isolates from poultry in the United States and those from a human urinary tract infection in Australia (Figure, panel B). Identical virulence factors and plasmid replicons were observed among 4 β-lactamase positive isolates and between 2 isolates missing the β-lactamase genes but carrying *mcr* colistin–resistance determinants. All 6 isolates had MDR profiles, phenotypically confirmed (data not shown except for those from colistin microdilution method) (Figure, panel B).

**Figure:**
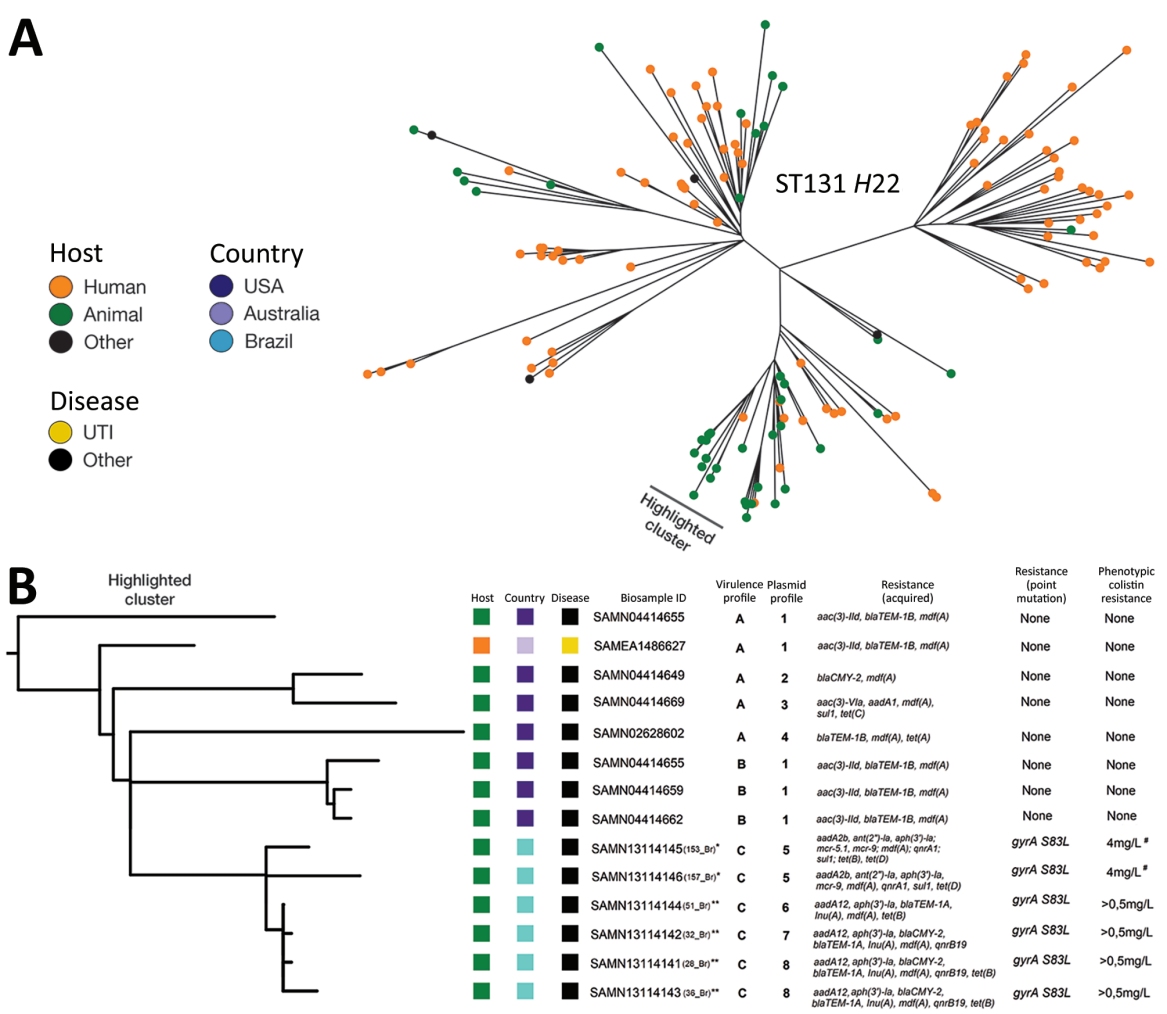
Phylogenetic analysis of *Escherichia coli* ST131-*H*22 isolates from poultry in Brazil and reference sequences. A) Unrooted phylogeny of 146 *E. coli* ST131-*H*22 isolates based on core genome single-nucleotide polymorphisms with the host origin outlined. The cluster containing closely related isolates to the 6 isolates from Brazil is highlighted. B) Rooted phylogeny of closely related isolates from retail meat with APEC and a human isolate with our 6 APEC isolates. The highlighted cluster includes a partial depiction of the tree including the data on host, country, and disease (urinary tract infection or other). Clusters containing the study’s isolates have their individual identification in parenthesis. Asterisks indicate farm origins (*, farm 1; **, farm 2). Virulence factors profiles are identified as groups A: *cvi/cva, ent, fimA-H, ibeA, irp1/2, iroN, iucD,iss, kpsM, ompA, tsh;* B: *cvi/cva, ent, fimA-H, ibeA, irp1/2, iroN, iucD, iss, kpsM, ompA*; and C: *cvi/cva, ent, fimA-H, fyuA, ibeA, irp1/2, iroN, iucD, iss, kpsM, ompA, tsh*. Plasmid profiles are identified by group: 1: IncFIB, IncFIC(FII), Incl1; 2: IncFIB, IncFIC(FII), IncFII, Incl1; 3: IncFIB, IncFIC(FII), IncFII, IncHI2, IncHI2A, Incl1; 4: IncFIB, IncFIC(FII), Incl1, IncN; 5: IncFIB, IncFIC(FII), IncFII, IncFII(pCoo), IncHI2, IncHI2A; 6: IncFIB, IncFIC(FII), IncFII, IncHI2, IncHI2A; 7: IncFIB, IncFII, Incl1, IncX1; and 8: IncFIB, IncFIC(FII), IncFII, Incl1, IncX1. Phenotypic colistin-resistance is indicated by the symbol # for the 2 colistin-resistance *mcr* genes positive isolates, showing resistance according to 2018 Clinical Laboratory Standards Institute (https://clsi.org/) clinical breakpoints. APEC, avian pathogenic *E. coli*; ID, identification; ST, sequence type; UTI, urinary tract infection.

The ST131-*H*22 lineage, while currently not as common as the *H*30 sublineage as a cause of community-acquired infections, does present a public health challenge because it colonizes poultry flocks, contaminating retail poultry products, and carries *mcr* colistin–resistance genes (*3*). The enormity and rapid growth of poultry production, in which many developing countries use antimicrobials extensively (*5*), and its zoonotic potential, make ST131-*H*22 worthy of specific attention (*2*).

Findings from our phylogenetic analyses of a global collection of ST131-*H*22 isolates from humans and poultry support findings from previous studies (*2*,*3*) and underscore the zoonotic potential of this virulent sublineage. Given that Brazil annually processes 13.8 million poultry products and exports 3.8 million kilograms (*4*), these findings warrant further examination to assess potential zoonotic spillover in Brazil and poultry-importing countries. Until such studies are conducted, the zoonotic potential of ST131-*H*22 in flocks in Brazil cannot be quantified.

The discovery of *mcr* mobile colistin resistance determinants in food animals has renewed attention to the potential risks of widespread antimicrobial use in livestock. In Latin America, *mcr-5* has been found in poultry in Paraguay (*9*). The description of the *mcr-9* homologue from humans in the United States and horses in Sweden has raised attention to another *mcr* gene with potential for global spread (*10*). Both *mcr* variants in this study, 153_Br and 157_Br, showed phenotypic resistance (*6*) and came from the same farm (Figure, panel B). Interestingly, 153_Br carried both *mcr-5.1* and *mcr-9* variants. These isolates may portend a more widespread problem within poultry flocks in Brazil.

Isolates from this study showed resistance to all of the World Health Organization’s highest priority critically important antimicrobial classes (Figure, panel B) (*8*). Analysis of the absence of tetracycline resistance (*tet*[B]/[D]) in 1 of our isolates (Figure, panel B) indicates partial plasmid loss (data not shown).

Use of colistin as a growth promoter in livestock was banned in Brazil in November 2016, although it continued being therapeutically used in poultry up to 2018 (*7*). Therefore, *mcr*-encoding *H*22 strains could be selected out of the population over time. Further restrictions will have to be implemented to combat the growing resistance of *E. coli* in poultry in Brazil to critically important antimicrobial drugs (*4*).

Our findings suggest that poultry in Brazil may serve as a reservoir for MDR extraintestinal pathogenic *E. coli* carrying mobile colistin-resistance determinants. These findings highlight the need for better antimicrobial stewardship and surveillance systems to determine the prevalence of MDR *E. coli* ST131-*H*22 in these poultry flocks and clarify the risks posed to domestic and international poultry consumers. 

Appendix Additional information on *mcr*-positive *Escherichia coli* ST131-*H*22 from poultry connected to international isolates, Brazil.

## References

[1] Manges AR. *Escherichia coli* and urinary tract infections: the role of poultry-meat. Clin Microbiol Infect. 2016;22:122–9. 10.1016/j.cmi.2015.11.010 26679924

[2] Liu CM, Stegger M, Aziz M, Johnson TJ, Waits K, Nordstrom L, et al. *Escherichia coli* ST131-*H*22 as a foodborne uropathogen. MBio. 2018;9:e00470–18. 10.1128/mBio.00470-18 30154256 PMC6113624

[3] Roer L, Overballe-Petersen S, Hansen F, Johannesen TB, Stegger M, Bortolaia V, et al. ST131 fim*H*22 *Escherichia coli* isolate with a *bla*_CMY-2_/IncI1/ST12 plasmid obtained from a patient with bloodstream infection: highly similar to *E. coli* isolates of broiler origin. J Antimicrob Chemother. 2019;74:557–60. 10.1093/jac/dky484 30496481

[4] Van Boeckel TP, Brower C, Gilbert M, Grenfell BT, Levin SA, Robinson TP, et al. Global trends in antimicrobial use in food animals. Proc Natl Acad Sci U S A. 2015;112:5649–54. 10.1073/pnas.1503141112 25792457 PMC4426470

[5] Doumith M, Day M, Ciesielczuk H, Hope R, Underwood A, Reynolds R, et al. Rapid identification of major *Escherichia coli* sequence types causing urinary tract and bloodstream infections. J Clin Microbiol. 2015;53:160–6. 10.1128/JCM.02562-14 25355761 PMC4290915

[6] Clinical and Laboratory Standards Institute. Performance standards for antimicrobial susceptibility testing; 28th informational supplement (M100–S28). Wayne (PA): The Institute; 2018.

[7] Brazil. Governmental Normative Instruction IN-45. Diario Oficial da Uniao. 2016. Nov 11 [cited 2020 Mar 20]. http://www.in.gov.br/materia/-/asset_publisher/Kujrw0TZC2Mb/content/id/22078290/do1-2016-11-30-instrucao-normativa-n-45-de-22-de-novembro-de-2016-22078259

[8] World Health Organization. Critically important antimicrobials for human medicine. 2011 [cited 2019 Oct 21]. http://apps.who.int/iris/bitstream/10665/77376/1/9789241504485%20eng.pdf

[9] Nesporova K, Jamborova I, Valcek A, Medvecky M, Literak I, Dolejska M. Various conjugative plasmids carrying the *mcr-5* gene in *Escherichia coli* isolates from healthy chickens in Paraguay. J Antimicrob Chemother. 2019;74:3394–7. 10.1093/jac/dkz317 31326998

[10] Börjesson S, Greko C, Myrenås M, Landén A, Nilsson O, Pedersen K. A link between the newly described colistin resistance gene mcr-9 and clinical Enterobacteriaceae isolates carrying bla_SHV-12_ from horses in Sweden. J Glob Antimicrob Resist. 2020;20:285–9. 10.1016/j.jgar.2019.08.007 31494305

