# Supplementary material for: mcr-Positive Escherichia coli ST131-H22 from Poultry in Brazil
Source: Emerg Infect Dis. 2020 Aug;26(8):1951–4. doi: 10.3201/eid2608.191724 (PMC7392447; doi:10.3201/eid2608.191724)
Supplement: Appendix — Additional information on mcr-positive Escherichia coli ST131-H22 from poultry connected to international isolates, Brazil. [file 19-1724-Techapp-s1.pdf]

Article DOI: https://doi.org/10.3201/eid2608.191724

mcr-Positive *Escherichia coli* ST131-*H*22 from Poultry Connected to International Isolates, Brazil

Appendix

Appendix Table. ST131-*H*22 genomic sequences (N = 140) obtained from GenBank and EnteroBase repositories and the 6 Brazilian poultry isolates from this study, with added metadata on host, continent, country of isolation, and disease, when available

Accession numbers (Biosample IDs)	Host	Continent	Country	Disease
SAMN04414658	Poultry	North America	United States	Other/food
SAMN04414651	Poultry	North America	United States	Other/food
SAMN04414654	Poultry	North America	United States	Other/food
SAMN04414650	Poultry	North America	United States	Other/food
SAMN04414646	Poultry	North America	United States	Other/food
SAMN04414652	Poultry	North America	United States	Other/food
SAMN04414653	Poultry	North America	United States	Other/food
SAMN04414671	Poultry	North America	United States	Other/food
SAMN04414657	Poultry	North America	United States	Other/food
SAMN04414698	Human	North America	United States	Urinary tract infection
SAMN04414684	Human	North America	United States	Urinary tract infection
SAMN04414818	Human	North America	United States	Urinary tract infection
SAMN04414670	Poultry	North America	United States	Other/food
SAMN04414668	Poultry	North America	United States	Other/food
SAMN04414799	Human	North America	United States	Urinary tract infection
SAMN04414648	Poultry	North America	United States	Other/food
SAMN04414645	Poultry	North America	United States	Other/food
SAMN04414660	Poultry	North America	United States	Other/food
SAMN04414655	Poultry	North America	United States	Other/food
SAMN04414659	Poultry	North America	United States	Other/food
SAMN04414662	Poultry	North America	United States	Other/food
SAMN04414649	Poultry	North America	United States	Other/food
SAMN04414669	Poultry	North America	United States	Other/food
SAMN04414647	Poultry	North America	United States	Other/food
SAMN04414661	Poultry	North America	United States	Other/food
SAMN04414667	Poultry	North America	United States	Other/food
SAMN04414678	Human	North America	United States	Urinary tract infection
SAMN04414779	Human	North America	United States	Urinary tract infection
SAMN04414780	Human	North America	United States	Urinary tract infection
SAMN04414676	Human	North America	United States	Urinary tract infection
SAMN04414790	Human	North America	United States	Urinary tract infection
SAMN04414797	Human	North America	United States	Urinary tract infection
SAMN04414700	Human	North America	United States	Urinary tract infection
SAMN04414767	Human	North America	United States	Urinary tract infection
SAMN04414798	Human	North America	United States	Urinary tract infection
SAMN04414738	Human	North America	United States	Urinary tract infection
SAMN04414761	Human	North America	United States	Urinary tract infection
SAMN04414793	Human	North America	United States	Urinary tract infection
SAMN04414844	Human	North America	United States	Urinary tract infection
SAMN04414827	Human	North America	United States	Urinary tract infection
SAMN04414822	Human	North America	United States	Urinary tract infection
SAMN04414810	Human	North America	United States	Urinary tract infection
SAMN04414816	Human	North America	United States	Urinary tract infection
SAMN04414831	Human	North America	United States	Urinary tract infection
SAMN04414809	Human	North America	United States	Urinary tract infection
SAMN04414848	Human	North America	United States	Urinary tract infection
SAMN07679510	Poultry	North America	United States	Other/food
SAMN04414665	Poultry	North America	United States	Other/food

Accession numbers (Biosample IDs)	Host	Continent	Country	Disease
SAMN04414663	Poultry	North America	United States	Other/food
SAMN02228508	Poultry	North America	United States	Colibacillosis
SAMN04992373	Poultry	North America	United States	Colibacillosis
SAMN02628602	Poultry	North America	United States	Other/food
SAMN02628600	Poultry	North America	United States	Other/food
SAMN02628555	Poultry	North America	United States	Other/food
SAMN02628549	Poultry	North America	United States	Other/food
SAMN02628547	Poultry	North America	United States	Other/food
SAMN02442811	Poultry	North America	United States	Other/food
SAMN02228511	Poultry	North America	United States	Colibacillosis
SAMN01885791	Human	Europe	Denmark	Urosepsis
SAMN01885786	Human	Europe	Denmark	Urosepsis
SAMN01885680	Human	Europe	Denmark	Urosepsis
SAMEA1486636	Human	North America	Canada	Septicemia
SAMEA1486663	Human	Europe	Spain	Urinary tract infection
SAMEA1486626	Human	Europe	Spain	Urosepsis
SAMEA1486672	Human	Europe	Spain	Urinary tract infection
SAMEA1486674	Human	Europe	Spain	Urinary tract infection
SAMEA1486627	Human	Oceania	Australia	Urinary tract infection
SAMN04159648	Human	Europe	Germany	Respiratory infection
SAMEA3712539	Human	Europe	Denmark	Other/not available
SAMN02603887*	Human	North America	United States	Reference strain for alignment
SAMN05170667	Human	Europe	United Kingdom	Other/not available
SAMN04273082	Human	Europe	United Kingdom	Septicemia
SAMN04273074	Human	Europe	Germany	Urinary tract infection
SAMN04273073	Human	Europe	Germany	Urinary tract infection
SAMN04357670	Human	Europe	United Kingdom	Septicemia
SAMEA2500548	Human	Europe	Spain	Abscess
SAMN05170071	Human	North America	United States	Urinary tract infection
SAMN05567350	Human	Oceania	Australia	Other/Feces
SAMEA3268827	Swine	Europe	Denmark	Other/Feces
SAMN02228459	Poultry	North America	United States	Other/not available
SAMN02228457	Poultry	North America	United States	Other/not available
SAMN02228456	Poultry	North America	United States	Other/not available
SAMN02442854	Poultry	North America	United States	Other/food
SAMN02801921	Human	North America	United States	Urinary tract infection
SAMN01885818	Human	Europe	Denmark	Urosepsis
SAMN01885841	Human	Europe	Denmark	Urosepsis
SAMN01885739	Human	Europe	Denmark	Urosepsis
SAMEA1486661	Human	Oceania	Australia	Urinary tract infection
SAMEA1486587	Human	Oceania	Australia	Urinary tract infection
SAMEA1486667	Human	Oceania	Australia	Urinary tract infection
SAMEA1486588	Human	Europe	United Kingdom	Urinary tract infection
SAMEA1486633	Human	Europe	United Kingdom	Urinary tract infection
SAMEA1486650	Human	Europe	United Kingdom	Urinary tract infection
SAMEA1486631	Human	Europe	United Kingdom	Urinary tract infection
SAMEA1486670	Human	Europe	Spain	Abdominal abscess
SAMEA1486601	Human	Europe	United Kingdom	Urinary tract infection
SAMEA1486662	Human	Europe	United Kingdom	Urinary tract infection
SAMN02228548	Human	Oceania	Australia	Other/not available
SAMN02228503	Human	North America	United States	Other/not available
SAMN02228498	Human	North America	United States	Other/not available
SAMN02228469	Human	North America	United States	Other/not available
SAMN02228455	Human	North America	United States	Other/not available
SAMN02228454	Human	Asia	India	Other/not available
SAMN02228453	Human	North America	United States	Other/not available
SAMN04159621	Human	Asia	Cambodia	Urinary tract infection
SAMN04159620	Human	Asia	Cambodia	Urinary tract infection
SAMN04159601	Human	Asia	Cambodia	Urinary tract infection
SAMN04159599	Human	Asia	Cambodia	Septicemia
SAMN04159570	Human	Europe	United Kingdom	Septicemia
SAMN04159569	Human	Europe	United Kingdom	Septicemia
SAMN02228503	Human	North America	Canada	Other/not available
SAMN02228496	Human	North America	United States	Other/not available
SAMN02228471	Human	North America	United States	Other/Environment
SAMN05163698	Human	Europe	United Kingdom	Other/not available
SAMN04273126	Human	Europe	United Kingdom	Urinary tract infection
SAMN04273123	Human	Europe	United Kingdom	Urinary tract infection

Accession numbers (Biosample IDs)	Host	Continent	Country	Disease
SAMN04273121	Human	Europe	Ireland	Urinary tract infection
SAMN04273096	Human	Europe	United Kingdom	Septicemia
SAMN04273095	Human	Europe	United Kingdom	Septicemia
SAMN04273093	Human	Europe	United Kingdom	Septicemia
SAMN04273090	Human	Europe	United Kingdom	Septicemia
SAMN04273088	Human	Europe	United Kingdom	Septicemia
SAMN04273067	Human	Europe	Germany	Urinary tract infection
SAMN04273066	Human	Europe	Germany	Urinary tract infection
SAMN04273061	Human	Europe	Germany	Urinary tract infection
SAMN03922994	Human	North America	United States	Septicemia
SAMN04357658	Human	Europe	United Kingdom	Septicemia
SAMEA1094703	Human	Europe	United Kingdom	Urinary tract infection
SAMN05170071	Human	North America	United States	Urinary tract infection
SAMD00044939	Human	Asia	Japan	Other/not available
SAMD00044931	Human	Asia	Japan	Other/not available
SAMEA3180298	Human	Europe	Germany	Other/not available
SAMN05567349	Human	Oceania	Australia	Feces
SAMN02228458	Human	North America	United States	Other/not available
SAMN02228472	Primate	North America	United States	Other/not available
SAMN04992249	Swine	North America	United States	Other/not available
SAMN02228471	Water	North America	United States	Other/Environment
SAMN05440287	Water	North America	United States	Other/Environment
SAMEA104402259	Human	South America	Brazil	Urinary tract infection
SAMEA104402296	Human	South America	Brazil	Urinary tract infection
SAMN13114141	Poultry	South America	Brazil	Colibacillosis
SAMN13114142	Poultry	South America	Brazil	Colibacillosis
SAMN13114143	Poultry	South America	Brazil	Colibacillosis
SAMN13114144	Poultry	South America	Brazil	Colibacillosis
SAMN13114145	Poultry	South America	Brazil	Colibacillosis
SAMN13114146	Poultry	South America	Brazil	Colibacillosis

*Reference strain *Escherichia coli* ST131 isolate JJ1886.
